# New global high-resolution centerlines dataset of selected river systems

**DOI:** 10.1016/j.dib.2018.09.016

**Published:** 2018-09-11

**Authors:** Zeenatul Basher, Abigail J. Lynch, William W. Taylor

**Affiliations:** a Center for Systems Integration and Sustainability, Michigan State University, East Lansing, MI 48823, USA; b National Climate Adaptation Science Center, United States Geological Survey, Reston, VA 20192, USA

## Abstract

We present the first high resolution (1:20,000) river centerlines shapefiles from 50 large rivers across the world. Rivers were selected based on the criteria of having more than 1000 km length and which have been reported to have a significant contribution to global fishery production. Since large rivers often span multiple countries, the degree of changes (i.e., anthropogenic or climate derived) varies from region to region. These high-resolution layers were developed to enable researchers to delineate accurate river length, from headwaters regions to their delta and assess or visualize the ongoing changes more accurately in these river systems. Further, these polylines could be used in coordination with satellite derived environmental or landscape variables for ecological research (e.g. predicting biodiversity, estimating biomass).

**Specification table**

**Table:** 

Subject area	Geography
Specific subject area	Rivers
Type of data	Figures, tables, Geographic Information Systems Shapefile (.shp)
How data was acquired	Drawn/Digitized using ESRI ArcGIS software
Data format	Raw digitized river polyline GIS shapefile.
Experimental factors	River centerlines were digitized following the NE GeoFeatures as baseline reference overlaid on top of satellite imagery.
Experimental features	Natural Earth GeoFeatures River centerlines were used for reference.
Data source location	Global
Data accessibility	Data is with this article and at https://www.sciencebase.gov/catalog/item/5a145fdde4b09fc93dcfd36c

**Value of the data**

• It is the first minimum 1:20,000 resolution spatial data of river centerlines, which can serve as a baseline data layer for future research in riverine ecosystems.
• This data will allow high-resolution delineation of river systems for geospatial analysis.
• No additional corrections or treatment were required as the polylines aligns with high-resolution satellite images and represent the exact length of the river from the headwaters to the delta.
• Have potential for use by an ecologist to identify changes in habitat and environmental change across different segments of the river (i.e., upstream to downstream).

## 1 Data

**Fig. 1:**
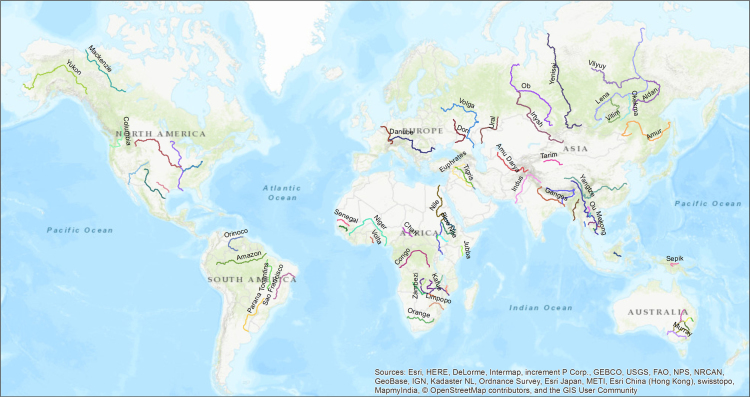
The global geographic distribution of 50 river centerlines. For a list of river names, see Table 1.

Three base data layers were used to derive the 1:20,000 resolution polygon shapefile: (1) a 300 m resolution raster waterbody dataset from European Space Agency (ESA) [1] (https://earth.esa.int/web/guest/data-access), (2) a 1:10 m vector polyline shapefile from the Natural Earth (NE) river and lakes centerlines [2], and (3) USGS 30′ Global Terrain Elevation Data [3], with an ArcGIS global topography basemap [4]. Although the new shapefile was digitized following the NE data layer, only two attributes (i.e., scale rank and river number) were transferred to new shapefile from the old datasets to add reference to a globally recognized dataset. Details about the fields included in the new vector high-resolution river centerline shapefile׳s features are given in (Fig. 1) Table 2.

**Table 1:** List of 50 rivers included in the new vector high-resolution river centerline shapefile.

**No.**	**Name**	**No.**	**Name**	**No.**	**Name**
1	Amazon	18	Irtysh	35	Rio Grande
2	Amu Darya	19	Jubba	36	Rufiji
3	Amur	20	Lena	37	Sao Francisco
4	Ayeyarwady	21	Limpopo	38	Senegal
5	Brahmaputra	22	Mackenzie	39	Sepik
6	Chao Phraya	23	Mahakam	40	Shatt Al Arab
7	Chari	24	Mekong	41	Tarim
8	Colorado	25	Mississippi	42	Tigris
9	Columbia	26	Missouri	43	Tocantins
10	Congo	27	Murray	44	Ural
11	Danube	28	Niger	45	Volga
12	Darling	29	Nile	46	Volta
13	Don	30	Ob	47	Yangtze
14	Euphrates	31	Orange	48	Yenisei
15	Gambia	32	Orinoco	49	Yukon
16	Ganges	33	Parana	50	Zambezi
17	Indus	34	Rhine

**Table 2:** Name and description of fields available in the new vector high-resolution river centerline shapefile (RiversCombo.shp).

**S. no.**	**Field name in shapefile**	**Field description**
1	Shape	ArcGIS Shapefile type field
2	Name	Name of the river
3	System	Name of the system the river belong to (e.g. Amazon)
4	name_alt	Alternative name of the river
5	scalerank	River scale referenced from Natural Earth dataset
6	rivernum	Unique river number referenced from Natural Earth dataset
7	Length_km	Length of the river (calculated with WGS84 World Mercator Projection in ArcGIS)

## 2 Experimental design, materials and methods

The new vector high-resolution river centerline shapefile dataset was digitized following the information extracted from base datasets as described above. Vector polylines of rivers were digitized manually by overlaying all three base map datasets (i.e., waterbody, basemap and Digital Elevation Model-DEM) and then drawing the polyline following the river flow direction assessed from the combination of all three datasets. First, we visually identified the start of rivers in the mountains and the end of rivers where the delta merges with the ocean. Polylines were then drawn (digitized) from headwaters of the river following river centerlines and contours down to its delta or river mouth. Locations where the topographic maps [4] or water body dataset [1] does not show any visible river body, DEM sinks were consulted to identify the centroid of river width and polylines were drawn following the linked pixels in DEM. Once the end of the river was reached in the digitization process, attributes of the rivers were transferred from the NE data to the new polyline. Every river polyline was assigned with its corresponding name, system, alternative names, scale, and length in kilometer (Table 2). The shapefile can be downloaded from https://www.sciencebase.gov/catalog/item/5a145fdde4b09fc93dcfd36c. Although there are shapefiles of river polylines at global scale exists, the major advancement of this layer is the improved resolution scale of 1:20,000 which will enable fisheries and aquatic scientist and managers to compare environmental data at ecologically relevant scales [5].

## References

[1] Pekel J.-F., Cottam A., Gorelick N., Belward A.S. (2016). High-resolution mapping of global surface water and its long-term changes. Nature.

[2] Natural Earth, Natural Earth Geo Features. Natural Earth Large Scale 1:10 m data of river+lakes centerlines V 2.0, 2012. [Online]. Available: 〈http://www.naturalearthdata.com/downloads/10m-physical-vectors/10m-rivers-lake-centerlines/〉. (Accessed 04 April 2016).

[3] USGS, USGS 30′ Global Terrain Elevation Data (GMTED2010). Data available from the U.S. Geological Survey, *See USGS* Visual Identity System Guidance, 2011. [Online]. Available: 〈https://topotools.cr.usgs.gov/gmted_viewer〉. (Accessed 12 July 2017).

[4] ESRI, Topographic [basemap]. Scale Not Given. ‘World Topographic Map,’” February 19, 2012*.*, [Online]. Available: 〈http://www.arcgis.com/home/item.html?Id=30e5fe3149c34df1ba922e6f5bbf808f〉. (Accessed 12 August 2017).

[5] Romulo C.L., Basher Z., Lynch A.J., Kao Y.-C., Taylor W.W. (2017). Assessing the global distribution of river fisheries harvest: a systematic map protocol. Environ. Evid..

